# A systematic review of congenital external ear anomalies and their associated factors

**DOI:** 10.3389/fped.2025.1520200

**Published:** 2025-04-24

**Authors:** Alejandro Acosta-Rodríguez, Sandra A. Reza-López, César R. Aguilar-Torres, Luis C. Hinojos-Gallardo, Dora V. Chávez-Corral

**Affiliations:** Department of Embryology, Facultad de Medicina y Ciencias Biomédicas, Universidad Autónoma de Chihuahua, Chihuahua, México

**Keywords:** auricle, ear anomalies, congenital malformations, external ear, review

## Abstract

**Objective:**

External ear anomalies may lead to conductive hearing loss with significant childhood disability, psychological distress, anxiety, social avoidance, and behavioral problems. The aim of this study is to compile and review published literature on the frequency of isolated and non-isolated external ear anomalies, their associated factors, and associated malformations/deformations in non-isolated cases.

**Methods:**

We conducted a systematic review in PubMed, Google Scholar, and Science Direct searching for any type of article (excluding reviews and meta-analyses) reporting isolated and non-isolated external ear anomalies in humans. Two authors extracted the information according to the main variables of interest according to PICO criteria. Details of studied population and main findings were also obtained (malformation type, unilateral or bilateral malformations and associated factors).

**Results:**

Twenty-six studies met eligibility criteria to be included in this review. Anotia/microtia was the most reported malformation, more frequently found in males, mostly unilateral; being the right ear the most affected, and more frequent in Hispanic population. Associated factors for external ear anomalies included parental age, maternal education, multiple pregnancies, high maternal body mass index and diabetes, pregnancy, and perinatal complications (low birth weight, prematurity, threatened abortion, etc.), twining, and chemical/drug exposure. The most reported malformations and syndromes associated with congenital external ear defects included: skull/face anomalies, cleft lip/palate, congenital heart defects, musculoskeletal malformations of skull, face and jaw, Treacher-Collins, OAVS (oculo-auriculo-vertebral spectrum), and trisomy 18, 13 and 21.

**Conclusion:**

Congenital external ear anomalies can occur isolated or associated with other malformations or syndromes. Environmental, socioeconomic, and cultural factors may partially explain the variation across populations for congenital external ear anomalies. Depending on their type and severity, they can lead to speech impediments and childhood disability, particularly in bilateral cases, highlighting the relevance of early detection and repair to avoid childhood disability.

## 1 Introduction

The incidence of ear malformations has been informed in approximately 1 per 3,800 newborns (1), while the incidence of external ear malformations occurs in 1 per 6,000 (2) to 6,830 newborns (3). Around 30% of them are associated with syndromes involving additional malformations and/or functional loss of organs and organ systems, such as Treacher-Collins, oculo-auriculo-vertebral spectrum or OAVS (also referred as Goldenhar syndrome or hemifacial microsomia), Crouzon, Apert, Klippel-Feil, Wildervanck, van der Hoeve-de-Kleyn, Albers-Schönberg, Patau, Edwards, Down, and 18q syndromes (4, 5). They can either affect the ear orientation, position (low set ear), size and/or shape of the auricle (microtia, cup ear, unfolded helix/Stahl ear); or result in a completely absent ear (anotia), while the middle ear can be atretic or hypoplastic. Minor malformations, such as ear tags, ear sinus and ear pits, may be also found (4). Atresia of the outer ear canal has been rarely observed in patients with a normal auricle (6).

Congenital anomalies of the external ear are genetic or acquired inborn anomalies of the auricle (4). They can be classified as deformations and malformations (7). A deformed ear is presented with fully developed components, with a misshaped auricle or pinna with intact cartilage and skin; while a malformed ear shows auricle alterations due to a partial or complete absence of cartilage and/or skin, because of underdevelopment during embryogenesis (8, 9). Most ear anomalies are acquired and originate from external forces applied to normal ear components *in utero* or postnatally (10), or by exposure to exogenous factors during the first trimester of pregnancy, such as: (a) infections, mainly viral, confirmed for rubella, cytomegalovirus, and herpes simplex virus; and possible for measles, mumps, hepatitis, poliomyelitis, chickenpox, Coxsackie virus and ECHO virus, and for toxoplasmosis, and syphilis; (b) chemical agents and medical drugs, such as thalidomide, quinine and aminoglycoside antibiotics, diphenylhydantoin, trimethadione, valproic acid, and excessively high doses of retinoic acid; (c) malnutrition and vitamin A deficiency during pregnancy; (d) Rh incompatibility; (e) hypoxia; (f) bleeding during the first trimester of pregnancy and disturbances of metabolism, such as diabetes. Environmental factors, including irradiation, atmospheric pressure changes, and noise exposure, should be also taken into consideration (1, 11–13). In many cases, however, the actual cause is unknown (14), because clinical and anamnestic data of exposure and exogenic influences are often missing or unclear (4).

Microtia/anotia is probably the most extensively studied external ear malformation. A recent metanalysis by Huang et al. (15), identified multiple risk factors with significant association for isolated microtia, including parental demographics, prenatal and perinatal characteristics (birth weight, chemical/medicinal exposure, infections), as well as familial history of ear malformations, among others, emphasizing the importance of identifying them to bring awareness and reinforce prevention. Expanding the scope for other congenital ear anomalies, that have received less attention and might also have consequences later in life, including non-isolated cases published reports, as well as exploring their associations with other congenital anomalies, could provide a more compelling review.

External ear anomalies could cause conductive hearing loss with a significant childhood disability, especially in bilateral cases (16, 17). Additionally, their effect on appearance may lead to psychological distress, anxiety, social avoidance, and behavioral problems (18). The purpose of this study was to compile and review published literature of isolated and non-isolated external ear anomalies, their characteristics, associated factors, and associated malformations/deformations in non-isolated cases.

## 2 Materials and Methods

### 2.1 Search strategy

A systematic literature review was conducted and reported following the Preferred Reporting Items for Systematic Reviews and Meta-Analyses (PRISMA) guidelines (19). Studies reporting risk factors for isolated and non-isolated congenital external ear anomalies were independently selected by two reviewers through a manual screening process in January 2025 using the advanced search tools from PubMed, Google Scholar and ScienceDirect databases.

In PubMed, the search terms used were “Outer Ear Malformations” OR “Outer Ear Defect” OR “External Ear Defect” OR “Microtia/Anotia” OR “Aural Atresia” contained in title/abstract, using the Boolean operators “NOT” to exclude “Reconstruction Surgery”, “Implants”, “Middle Ear”, “Inner/Internal Ear” and “Deafness”. In Google Scholar, the search terms were “Outer Ear Malformations” OR “Outer Ear Defects” OR “Microtia/Anotia” OR “Aural Atresia” in title, and without the words “Deafness, Mice, Rats, Inner [ear] & Internal [ear]”. Finally, in ScienceDirect, the terms “Ear Malformations” OR “Microtia/Anotia” OR “Aural Atresia” in the title, abstract or author-specified key words were searched, using the same restriction words than those used in Google Scholar.

The filters applied in PubMed were article type (any type, excluding reviews and meta-analyses) and species (humans); in ScienceDirect they were article type (any type, excluding reviews and meta-analyses) and subject area (medicine); in Google Scholar, articles were manually selected excluding review articles and meta-analyses. In all databases, filters for year (2000 to 2025) and language (English, Spanish, French, Italian and Portuguese) were also applied. Duplicates were manually removed.

### 2.2 Eligibility criteria

Research articles that included any type of external ear anomalies, isolated and non-isolated, and reporting association measures with risk factors or results of comparison tests (relative risk, odds ratio, and *P*-value), in humans were included. Articles referring only to the middle and/or inner ear malformations, tumors, trauma, surgery, animal models, and specific syndromes were excluded, as well as case reports.

### 2.3 Data extraction

The title, authors, year of publication, language, and place of publication were obtained and registered. The information on the main variables of interest was extracted according to PICO (Population, Intervention, Comparison and Outcome) criteria as follows: [1] Population (individuals diagnosed with any outer ear anomalies); [2] Exposure -instead of intervention- (at least one identifiable risk factor for external ear anomalies); [3] controls in case control studies (individuals with unknown outer ear malformations and/or history of exposure), and [4] Outcomes: main findings, both descriptive (malformation type, unilateral or bilateral malformations), associated factors and association measures (relative risk, RR; odds ratio, OR) or those reporting a *P*-value for group comparisons, and associated deformations/malformations and syndromes, when reported.

## 3 Results

**Figure 1:**
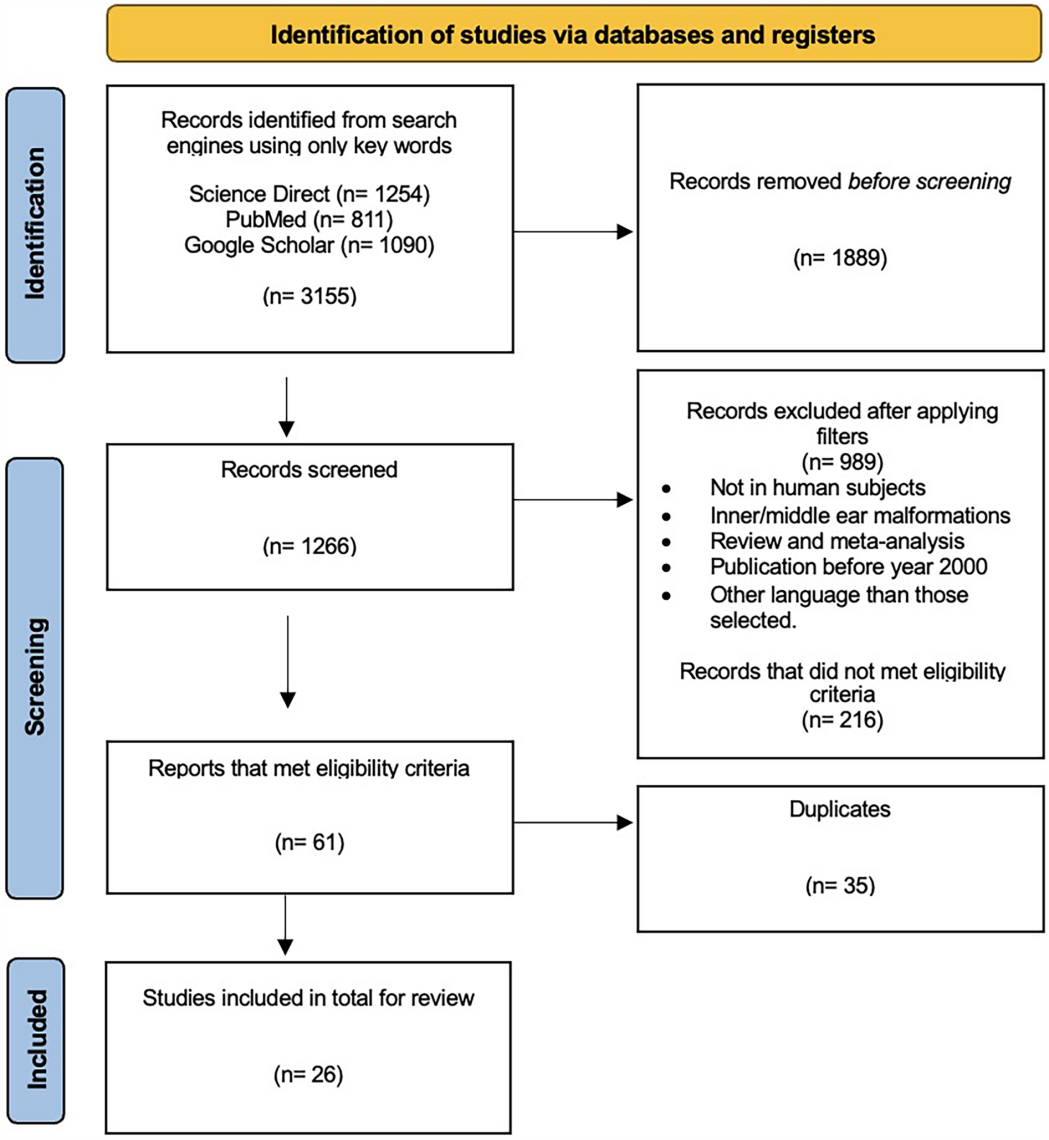
Flow diagram of publication selection process in accordance with Preferred Reporting Items for Systematic Reviews and Meta-Analyses (PRISMA) guidelines. Source: Page et al. (19).

A total of 1,266 studies were identified −880 from Google Scholar, 158 from PubMed, and 228 from ScienceDirect. After screening of titles and abstracts, removing duplicates, and verifying eligibility criteria, 26 articles remained to be included in this review (Figure 1).

Sixteen studies were case-control studies (20–35), eight were retrospective reviews (36–43), one was a cohort study (44), and other was a cross-sectional study (45). Ten were studies from the U.S.A (22, 23, 25, 26, 29, 31, 33, 36–38), ten from China (21, 27, 28, 30, 32, 39–43), three from Mexico, Colombia, and South America (20, 24, 34), and the rest from Israel, Japan, and South Korea [*n* = 1, each] (35, 44, 45), as shown in Table 1. All studies were published in English.

**Table 1:** Characteristics of studies included in the review.

First author	Place	Study design
Bader et al. (44)	Israel	Cohort
Shaw et al. (36)	U.S.A.	Retrospective review
Forrester and Merz (37)	U.S.A.	Retrospective review
Canfield et al. (38)	U.S.A.	Retrospective review
Garcia et al. (20)	Colombia	Case-control
Zhang et al. (21)	China	Case-control
Ma et al. (22)	U.S.A	Case-control
Wu et al. (43)	China	Retrospective review
Lee et al. (35)	South Korea	Case-control
Ma et al. (23)	U.S.A.	Case-control
Yamauchi et al. (45)	Japan	Cross-sectional
Luquetti et al. (24)	South America	Case-control
Van Bennekom et al. (25)	U.S.A.	Case-control
Hoyt et al. (26)	U.S.A.	Case-control
Li et al. (28)	China	Case-control
Deng et al. (39)	China	Retrospective review
Liu et al. (27)	China	Case-control
Ryan et al. (29)	U.S.A.	Case-control
Guo et al. (40)	China	Retrospective review
Chen et al. (30)	China	Case-control
Sheehan et al. (31)	U.S.A.	Case-control
Sun et al. (41)	China	Retrospective review
Yu et al. (32)	China	Case-control
Schraw et al. (33)	U.S.A.	Case-control
Ibarra et al. (34)	Mexico	Case-control
Zhou et al. (42)	China	Retrospective review

The reported external ear anomalies and their frequency by sex, laterality, and ethnicity are shown in Table 2. The anotia/microtia was the most studied malformation (20–31, 33–43, 45). A study reported low set ears as the most common malformation (44), and two studies reported the frequency of deformations: Stahl ear and preauricular tags (34, 44).

**Table 2:** External ear anomalies and their association with sex, and laterality.

First Author	Anomalies (*n*)%	Prevalence by sex (%)	Side (%)
Bader et al. (44)	Auricular mild errors of morphogenesis (*n* = 1,342)	Male 47.1%	NR
	Deformation 57.35%: Stahl Ear 13.7%		Bilateral 78.6%
	Malformation 13.4%: Low set ear 2.96%		Bilateral 61.69%
Shaw et al. (36)	Microtia/Anotia (*n* = 549)	NR	NR
	Non-isolated 70.86%	Male 56%	Unilateral 72.75%
	Isolated 29.1%	Male 57.5%	Unilateral 94%
Forrester and Merz (37)	Microtia (*n* = 109) 90.8% Anotia (*n* = 10) 8.3%	Male 62.5%	Unilateral 79.8% (Right ear 64%)
Canfield et al. (38)	Microtia (*n* = 698) 94.1% Anotia (*n* = 44) 5.9%	Male 56.7%	Unilateral 77%
Garcia-Reyes et al. (20)	Isolated microtia (*n* = 27)	Male 62.96%	Unilateral 85.1% (right ear 62.9%)
Zhang et al. (21)	Isolated microtia (*n* = 121)	Male 78.5%	NR
Ma et al. (22)	Microtia (*n* = 420) Isolated 73% Non-isolated 27%	NR	NR
Wu et al. (43)	Microtia/Anotia (*n* = 345) Isolated 56.52% Non-isolated 43.48%	Male 72.75%	Unilateral 92% (right ear 55.24%)
Lee et al. (35)	Microtia (*n* = 374) Isolated 65% Non-isolated 35%	Male 67.3%	Unilateral 93.3% (right ear 53.2%)
Ma et al. (23)	Microtia (*n* = 382) Isolated 75% Non-isolated 25%	NR	NR
Yamauchi et al. (45)	Microtia (*n* = 428)	Male 61%	Unilateral 90% (right ear 59%)
Luquetti et al. (24)	Isolated microtia (*n* = 1,194)	Male 56.6%	Unilateral 82% (right ear 65.6%)
Van Bennekom et al. (25)	Microtia (*n* = 421) Isolated 72% Non-isolated 28%	Male 60% Male 58%	NR
Hoyt et al. (26)	Microtia/Anotia (*n* = 507) Isolated 71%	Male 59.5%	NR
Li et al. (28)	Microtia (*n* = 911) Isolated 69.5% Non-isolated 30.5%	Male 69.7%	Unilateral 74% (right ear 57.2%)
Deng et al. (39)	Microtia/Anotia (*n* = 1,933) Isolated 73.41% Non-isolated 26.59%	Male 58.56%	NR
Liu et al. (27)	Severe microtia/atresia (*n* = 322)	Male 68.6%	Unilateral 80.7% (right ear 54%)
Ryan et al. (29)	Microtia/Anotia (*n* = 699) Non-isolated 31% Isolated 69%	Male 57.2%	Unilateral 87%
Guo et al. (40)	Severe microtia (*n* = 965) Isolated 89.8% Non-isolated 40.6%	Male 65.2%	Unilateral 83.1% (right ear 52%)
Chen et al. (30)	Microtia (*n* = 293)	Male 73.7%	NR
Shehan et al. (31)	Microtia/Anotia (*n* = 523)	Male 55.98%	NR
Sun et al. (41)	Microtia (*n* = 115)	Male 59.13%	NR
Yu et al. (32)	Congenital ear malformations (*n* = 1,676)	Male 57%	NR
Schraw et al. (33)	Microtia (*n* = 1,236) 93.5% Anotia (*n* = 86) 6.5%	Male 57.5%	Unilateral 88.88% (right ear 64.8%)
Ibarra et al. (34)	Isolated Microtia (*n* = 167) Preauricular tag (*n* = 656)	NR	NR
Zhou et al. (42)	Congenital malformations of external ear (*n* = 1,227) Microtia/Anotia (*n* = 185)	Male 58.2% Male 63.7%	NR

NR, not reported.

From the selected studies, twenty-three reported subject sex, showing greater occurrence of ear anomalies in male population (20, 21, 24–33, 35–45). Fourteen studies reported laterality (20, 24, 27–29, 33, 35–38, 40, 43–45), from which thirteen reported that congenital external ear anomalies were mostly unilateral, and ten being the right ear the most affected (20, 24, 27, 28, 33, 35, 37, 40, 43, 45). Only one study reported higher prevalence for bilateral anomalies (44). Finally, eleven studies classified cases by ethnic background: nine observed higher frequency among Hispanic population, compared to non-Hispanic Caucasians and African Americans (22, 23, 25, 26, 29, 31, 33, 36, 38), and two studies focused on Jewish, Arab (44), Pacific Islanders and Filipino descendants (37) Table 3.

**Table 3:** External ear anomalies and their association with ethnicity.

First author and year	Ethnicity
Bader et al. (44)	Ashkenazi (597/1,368) 43.6% and Sephardic Jewish (378/887) 42.6% Muslim Arab (194/418) 46.4% and Christian Arab (78/190) 41.1% Christian non-Arab (32/84) 38.1% Druze (50/129) 38.8% Ethiopian Jewish (5/11) 45.5% Other (8/20) 40%
Shaw et al. (36)	Non-isolated cases (*n* = 389) White, non-Hispanic (72) 18.5%; U.S. born Hispanic (69) 17.74%; foreign born Hispanic (185) 47.6%; Black (20) 5.1%; and Asian (20) 5.1% Isolated cases (*n* = 160) White, non-Hispanic (13) 8.12%; US-born Hispanic (30) 18.75%; Hispanic (100) 62.5%; Black (1) 0.6%; and Asian (10) 6.25%
Forrester and Merz (37)	Pacific Islander (41/106) 39% Filipino (28/106) 26.4%
Canfield et al. (38)	White, non-Hispanic (183) 24.7% Black, non-Hispanic (34) 4.6% Hispanic (508) 68.5%: born in Mexico 34%, US-born 28.4%, other 6.1%
Ma et al. (22)	White, non-Hispanic (154) 36.7% Black, non-Hispanic (12) 2.9% Hispanic (222) 52.7% Other (29) 6.9%
Ma et al. (23)	White, non-Hispanic (144) 37.7% Black, non-Hispanic (9) 2.4% Hispanic (199) 52.1% Other (27) 7.1%
Van Bennekom et al. (25)	Any microtia (*n* = 421): White, non-Hispanic: 37% Black, non-Hispanic: 3% Hispanic: 53% Other: 7% Isolated microtia (*n* = 304): White, non-Hispanic: 33% Black, non-Hispanic: 2% Hispanic: 56% Other: 8%
Hoyt et al. (26)	White, non-Hispanic: (119) 33.9% Black, non-Hispanic: (9) 2.6% Hispanic: (194) 55.3% Other: (29) 8.3%
Ryan et al. (29)	Hispanic (376) 53.8% White, non-Hispanic (242) 34.6% Black, non-Hispanic (27) 3.9% Other (54) 7.7%
Shehan et al. (31)	White (180) 34.41% Black (26) 5.08% Hispanic (191) 36.58% Asian or pacific islander (29) 5.58% Native American (5) 1.06% Other (36) 6.9%
Schraw et al. (33)	All cases (1,322): White, non-Hispanic 18.1%; Black, non-Hispanic 3%; Hispanic 73.8%; other non-Hispanic 3.9% Isolated cases (982): White, non-Hispanic 18.5%; Black, non-Hispanic 2.3%; Hispanic 74.7%; other non-Hispanic 3.9% Non-isolated cases (340): White, non-Hispanic 16.8%; Black, non-Hispanic 5%; Hispanic 71.2%; other non-Hispanic 4.1%

### 3.1 Associated factors

Some authors associated congenital anomalies of the external ear with parental age, race/ethnicity, education level, residential area, infant sex, multiple pregnancies, twining, abortions, obesity, pregnancy and perinatal complications, and chemical/medication exposure. Risk factors by characteristics (demographics) and parental health behaviors are shown in Table 4, risk factors by pregnancy characteristics and parental clinical features are shown in Table 5, and risk factors of studies reporting microtia/anotia compared by all, isolated, and non-isolated cases are shown in Table 6.

**Table 4:** Risk factors for external ear malformations classified by demographics and parental health behavior.

Author, year	Risk factors	OR (95% CI), *n* (prevalence)
Parental age		
Maternal age <25		
Hoyt, 2014 (26)	U.S born Hispanics vs. White, non-Hispanic	aOR = 4.43, 95% CI (2.17–9.02)^a^
	Foreign born Hispanics vs. White, non-Hispanic	aOR = 5.90, 95% CI (2.82–12.33)^a^
Yamauchi, 2012 (45)	vs. mean age of controls	*n* = 34, 8.5%; *p* < 0.01^f^
Maternal age 25–29		
Hoyt, 2014 (26)	Foreign born Hispanics vs. White, non-Hispanic	aOR = 4.67, 95% CI (2.12–10.30)^a^
Yu, 2022 (32)	Maternal age <30 compared with controls	*n* = 1080, 64.4%; *p* < 0.001^f^
Yamauchi, 2012 (45)	vs. mean age of controls	*n* = 149, 39%; *p* < 0.01^f^
Maternal age 30–34		
Canfield, 2009 (38)	vs. maternal age 25–29	aOR = 1.35, 95% CI (1.04–1.75)^b^
Yamauchi, 2012 (45)	vs. mean age of controls	*n* = 150, 40%; *p* < 0.01^f^
Maternal age ≥35		
Hoyt, 2014 (26)	Foreign born Hispanics vs. White, non-Hispanic	aOR = 3.91, 95% CI (1.23–12.46)^a^
Liu, 2018 (27)	vs. maternal age <26	OR = 8.17, 95% CI (3.78–17.6)
Yamauchi, 2012 (45)	vs. mean age of controls	*n* = 45, 12%; *p* < 0.01^f^
Paternal age >35		
Liu, 2018 (27)	vs. paternal age <26	OR = 6.69, 95% CI (3.64–12.29)
Maternal race/ethnicity		
Shehan, 2022 (31)	Hispanic vs. White, non-Hispanic	OR = 2.76, 95% CI (2.28–3.34)
	Native American vs. White, non-Hispanic	OR = 2.21, 95% CI (1.02–4.78)
	Other vs. White, non-Hispanic	OR = 1.51, 95% CI (1.08–2.10)
	Black vs. White, non-Hispanic	OR = 0.50, 95% CI (0.34–0.72)
Hoyt, 2014 (26)	Hispanic vs. White, non-Hispanic	*n* = 194, 55.3%, *p* < 0.01^f^
	Black, non-Hispanic vs. White, non-Hispanic	*n* = 9, 2.6%, *p* < 0.01^f^
Parental education level		
Maternal <High school		
Canfield, 2009 (38)	vs. >High school	aOR = 2.98, 95% CI (1.17–8.50)^b^
Zhang, 2009 (21)	vs. >High school	OR = 3.00, 95% CI (1.672–5.381)
Hoyt, 2014 (26)	U.S born Hispanics vs. White, non-Hispanic	aOR = 4.93, 95% CI (1.38–17.61)^a^
	Foreign born Hispanics vs. White, non-Hispanic	aOR = 8.79, 95% CI (2.52–30.60)^a^
Yu, 2022 (32)	vs. >High school	*n* = 1121, 66.9%; *p* < 0.001^f^
Maternal ≥High school		
Canfield, 2009 (38)	High school graduate vs. >High school	aOR = 3.97, 95% CI (1.68–10.69)^b^
Hoyt, 2014 (26)	U.S. born Hispanics vs. White, non-Hispanic	aOR = 2.30, 95% CI (1.25–4.22)^a^
	Foreign born Hispanics vs. White, non-Hispanic	aOR = 3.76, 95% CI (2.10–6.72)^a^
Paternal <High school		
Zhang, 2009 (21)	vs. >High school	OR = 5.249, 95% CI (2.464–11.179)
Maternal employment		
Luquetti, 2013 (24)	Outside of home vs. housewife	aOR = 1.3, 95% CI (1.1–1.5)^c^
Household income		
Hoyt, 2014 (26)	<10,000 vs. White, non-Hispanic	aOR = 2.83, 95% CI (1.07–7.52)^a^
	10,000–19,000 vs. White, non-Hispanic	aOR = 3.35, 95% CI (1.37–8.21)^a^
	20,000–39,999 vs. White, non-Hispanic	aOR = 5.06, 95% CI (2.37–10.80)^a^
	≥40,000 vs. White, non-Hispanic	aOR = 3.21, 95% CI (1.25–8.25)^a^
Parental resident area		
Zhang, 2009 (21)	Rural vs. urban areas	OR = 8.286, 95% CI (3.782–18.152)
Zhou, 2024 (42)	Urban vs. rural areas	OR = 1.45, 95% CI (1.29–1.62)
Lee, 2012 (35)	Urban vs. rural areas	OR = 0.174, 95% CI (0.047–0.653)
Altitude		
Ibarra, 2024 (34)	1,511–2,426 m vs. low altitude (≤1,499 m)	OR = 1.60, 95% CI (1.34–1.92)
Environmental pollution		
Zhang, 2009 (21)	NS	OR = 7.0, 95% CI (2.088–23.468)
Chen, 2022 (30)	NS	OR = 1.82, 95% CI (1.19–2.70)
Parental chemical exposure		
Maternal exposure		
Zhang, 2009 (21)	NS	OR = 4.764, 95% CI (1.659–13.680)
Li, 2014 (28)	NS	aOR = 2.77, 95% CI (1.78–4.32)^d^
Liu, 2018 (27)	Heavy metals	OR = 4.6, 95% CI (0.99–21.46)
Chen, 2022 (30)	NS	OR = 3.20, 95% CI (1.17–8.73)
Yu, 2022 (32)	SO2 before conception	aOR = 1.93, 95% CI (1.43–2.59)^e^
	SO2 after conception	aOR = 1.63, 95% CI (1.22–2.18)^e^
Paternal exposure		
Chen, 2022 (30)	Dust	OR = 3.42, 95% CI (1.80–6.50)
	Heavy metals	OR = 2.92, 95% CI (1.51–5.62)
Drinking		
Lee, 2012 (35)	Positive drinking vs. non-drinkers	OR = 4.065, 95% CI (1.764–9.212)
Smoking ≥ 1 cigarette daily		
Maternal		
Luquetti, 2013 (24)	Foreign born Hispanic vs. White, non-Hispanic	aOR = 1.7, 95% CI (1.1–2.6)^c^
Hoyt, 2014 (26)	Positive smoking vs. non-smokers	aOR = 6.95, 95% CI (2.58–18.71)^a^
Paternal		
Chen, 2022 (30)	Positive smoking vs. non-smokers	OR = 2.07, 95% CI (1.44–2.96)
Pet contact		
Zhang, 2009 (21)	Contact vs. no contact	OR = 4.789, 95% CI (1.831–12.578)
Family history of congenital ear malformation		
Garcia, 2009 (20)	Positive history vs. no history	OR = 2.93, 95% CI (1.01–8.48)
Luquetti, 2013 (24)	Positive history vs. no history	aOR = 18.1, 95% CI (7.9–41.4)^c^

Isolated cases: anotia/microtia with no other structural anomaly diagnosis, non-isolated cases: anotia/microtia with the presence of other structural anomalies (excluding chromosomal anomalies), NS, not specified; OR, odds ratio; aOR, adjusted odds ratio; CI, confidence interval.

^a^ Adjusted for pregnancy BMI, age, education, folic acid, gestational diabetes, smoking, alcohol intake, annual household income, and study center.

^b^ Adjusted for maternal age, race/ethnicity, border residence, maternal birthplace, maternal education, year of infant birth, and infant sex.

^c^ Adjusted by sex, maternal age, hospital, and year of birth.

^d^ Adjusted by gender, age, region, syndrome, and family history.

^e^ Adjusted for maternal age, season of conception, gravidity, parity, maternal education, nitrogen dioxide and particulate matter with an aerodynamic diameter ≤10 μm exposure levels during the same period.

^f^ Risk factor prevalence.

**Table 5:** Risk factors for external ear malformations classified by pregnancy and parental clinical features.

Author, year	Risk factors	OR 95% (CI), *n* (prevalence)^e^
Infant sex		
Male		
Hoyt, 2014 (26)	U.S. born Hispanics vs. White, non-Hispanic	aOR = 2.09, 95% CI (1.17–3.73)^a^
	Foreign born Hispanics vs. White, non-Hispanic	aOR = 3.75, 95% CI (2.21–3.68)^a^
Canfield, 2009 (38)	vs. female infants	aOR = 1.27, 95% CI (1.06–1.52)^b^
Zhou, 2024 (42)	vs. female infants	OR = 1.57, 95% CI (1.16–2.12)
Bader, 2004 (44)	vs. female infants	*n* = 760, 47.1%; *p* < 0.0001^e^
Yu, 2022 (32)	vs. female infants	*n* = 956, 57%; *p* < 0.001^e^
Sun, 2022 (41)	vs. female infants	*n* = 68, 59%; *p* < 0.05^e^
Yamauchi, 2012 (45)	vs. female infants	*n* = 230, 61%; *p* < 0.01^e^
Female		
Hoyt, 2014 (26)	U.S. born Hispanic vs. White non-Hispanic	aOR = 2.86, 95% CI (1.44–5.68)^a^
	Foreign born Hispanic vs. White non-Hispanic	aOR = 4.76, 95% CI (2.55–8.89)^a^
Prematurity		
Forrester, 2005 (37)	vs. ≥30 weeks	OR = 2.27, 95% CI (1.49–3.40)
Shehan, 2022 (31)	vs. full-term population	OR = 2.19, 95% CI (1.78–2.69)
Yu, 2022 (32)	vs. full-term population	*n* = 102, 6.1%; *p* < 0.001^e^
Bader, 2004 (44)	vs. full-term population	*n* = 101, 53.2%; *p* = 0.014^e^
Gestational weight		
Low birth weight		
Forrester, 2005 (37)	vs. ≥2500 gr	OR = 3.35, 95% CI (2.04–5.30)
Garcia, 2009 (20)	vs. ≥2,500 gr	OR = 3.25, 95% CI (1.11–9.58)
Yu, 2022 (32)	NS	*n* = 89, 5.3%; *p* < 0.001^e^
Bader, 2004 (44)	NS	*n* = 64, 48.9%; *p* = 0.044^e^
Large for gestational age		
Bader, 2004 (44)	NS	*n* = 108, 49.8%; *p* < 0.0001^e^
Maternal BMI		
Hoyt, 2014 (26)	Pregnancy BMI 18.5 ≤ 24.9	
	U.S. born Hispanic vs. White, non-Hispanic	aOR = 2.37, 95% CI (1.30–4.34)^a^
	Foreign born Hispanic vs. White, non-Hispanic	aOR = 3.11, 95% CI (1.82–5.33)^a^
	Pregnancy BMI 25 ≤ 29.9	
	Foreign born Hispanic vs. White, non-Hispanic	aOR = 4.83, 95% CI (1.95–11.97)^a^
	Pregnancy BMI ≥ 30	
	U.S. born Hispanic vs. White, non-Hispanic	aOR = 3.67, 95% CI (1.29–10.41)^a^
	Foreign born Hispanic vs. White, non-Hispanic	aOR = 11.87, 95% CI (4.31–32.71)^a^
Gestational diabetes		
Hoyt, 2014 (26)	Foreign born Hispanics vs. White, non-Hispanic	aOR = 6.07, 95% CI (1.09–33.69)^a^
Multiple pregnancies		
Forrester, 2005 (37)	NS vs. single birth	OR = 3.72, 95% CI (1.66–7.33)
Luquetti, 2013 (24)	Parity 2–7 vs. primipara	aOR = 1.5, 95% CI (1.2–1.8)^c^
	Parity ≥8 vs. primipara	aOR = 2.8, 95% CI (1.6–5.2)^c^
Yu, 2022 (32)	Gravidity ≥2 vs. primipara	*n* = 758, 45.2%; *p* < 0.001^e^
	Parity ≥2 vs. primipara	*n* = 328, 19.6%; *p* < 0.001^e^
Chen, 2022 (30)	Gravidity ≥1	OR = 2.42, 95% CI (1.89–3.09)
	Parity ≥1	OR = 1.57, 95% CI (1.14–2.15)
Shehan, 2022 (31)	Non-singletons vs. singletons	OR = 4.39, 95% CI (6.07–34.12)
Parental chronic diseases		
Maternal		
Luquetti, 2013 (24)	NS	aOR = 1.3, 95% CI (1.0–1.7)^c^
Chen, 2022 (30)	NS	OR = 2.25, 95% CI (1.25–4.05)
Shehan, 2022 (31)	Diabetes	OR = 4.64, 95% CI (2.06–10.46)
Paternal		
Chen, 2022 (30)	NS	OR = 4.38, 95% CI (2.03–9.43)
Maternal infections during pregnancy		
Zhang, 2009 (21)	During pregnancy	OR = 7.714, 95% CI (3.510–16.953)
	During first trimester	OR = 7.469, 95% CI (3.324–16.784)
	After first trimester	OR = 3.108, 95% CI (1.180–8.185)
Wu, 2010 (43)	Viral infection (influenza or Measles)	*n* = 73, 48.21% *p* = 0.0474^e^
Luquetti, 2013 (24)	Acute diseases during pregnancy	aOR = 1.4, 95% CI (1.2–1.6)^c^
	Cold-like symptoms	aOR = 2.2, 95% CI (1.2–3.9)^c^
Li, 2014 (28)	Cold-like symptoms	aOR = 3.12, 95% CI (2.30–4.25)^d^
	Inflammatory infection	aOR = 3.56, 95% CI (2.07–6.13)^d^
Liu, 2018 (27)	Viral illness	OR = 1.933, 95% CI (1.148–3.256)
Chen, 2022 (30)	Genital infection	OR = 16.75, 95% CI (6.11–45.93)
	Urinary tract infection	OR = 7.25, 95% CI (2.55–20.62)
	Fever	OR = 2.27, 95% CI (1.23–4.16)
Shehan, 2022 (31)	Acute respiratory infection	OR = 2.23, 95% CI (1.35–3.68)
	Infectious or parasitic diseases	OR = 2.50, 95% CI (1.40–4.47)
Maternal medicatio		
Zhang, 2009 (21)	During pregnancy (medication non specified)	OR = 3.400, 95% CI (1.680–6.882)
	During first trimester (medication non specified)	OR = 6.618, 95% CI (2.452–17.857)
Lee, 2012 (35)	NS	OR = 6.077, 95% CI (2.413–15.307)
Luquetti, 2013 (24)	Analgesics	aOR = 2.2, 95% CI (1.4–3.4)^c^
	Antibiotics	aOR = 1.6, 95% CI (1.1–2.5)^c^
	Antiemetics	aOR = 4.5, 95% CI (1.0–21.4)^c^
	Antihypertensives	aOR = 3.6, 95% CI (1.0–13.7)^c^
	Antispasmodics	aOR = 2.8, 95% CI (1.2–6.5)^c^
	Drugs for functional bowel disease	aOR = 3.6, 95% CI (1.5–8.5)^c^
	Progestogens/estrogens combinations	aOR = 6.1, 95% CI (2.1–18.1)^c^
Liu, 2018 (27)	NSAIDs	OR = 2.576, 95% CI (1.079–6.148)
	Progesterone	OR = 2.71, 95% CI (1.49–4.95)
	Traditional Chinese medicine	OR = 2.86, 95% CI (1.36–6.00)
Chen, 2022 (30)	Teratogenic drug use	OR = 5.31, 95% CI (3.11–9.06)
	Oral contraceptives	OR = 2.14, 95% CI (1.14–4.04)
Folate supplementation during pregnancy		
No folate intake		
Hoyt, 2014 (26)	U.S. born Hispanics vs. White, non-Hispanic	aOR = 2.74, 95% CI (1.55–4.84)^a^
	Foreign born Hispanics vs. White, non-Hispanic	aOR = 5.22, 95% CI (3.08–8.87)^a^
Adecuate folate intake		
Li, 2014 (28)	vs. no intake	aOR = 0.35, 95% CI (0.27–0.47)^d^
Chen, 2022 (30)	vs. no intake	OR = 0.25, 95% CI (0.16–0.38)
Threatened abortion		
Lee, 2012 (35)	Yes vs. no	OR = 3.828, 95% CI (1.093–13.412)
Liu, 2018 (27)	Yes vs. no	OR = 4.066, 95% CI (2.36–7.0)
Chen, 2022 (30)	Yes vs. no	OR = 1.91, 95% CI (1.25–2.91)
History of spontaneous abortion		
Li, 2014 (28)	Yes vs. no	aOR = 5.16, 95% CI (2.88–9.24)^d^
Liu, 2018 (27)	Yes vs. no	OR = 6.49, 95% CI (2.16–19.53)
Wu, 2010 (43)	Yes vs. no	*n* = 15, 31.73%; *p* = 0.0309^e^
Abnormal pregnancy history		
Chen, 2022 (30)	NS	OR = 4.86, 95% CI (3.23–7.29)
	Vaginal bleeding	OR = 2.16, 95% CI (1.42–3.28)
Liu, 2018 (27)	Anemia during first trimester	OR = 1.902, 95% CI (1.026–3.526)
Garcia, 2009 (20)	NS	OR = 2.39, 95% CI (1.01–5.67)
Immunization before pregnancy		
Lee, 2012 (35)	Rubella	OR = 0.214, 95% CI (0.115–0.400)
Guo, 2021 (40)	Predictive nomogram for maternal age, miscarriage frequency, virus infection, anemia, progesterone, paternal alcohol intake and topography of living areas	(*n* = 965) C-index = 0.755, 95% CI, 0.703–0.807, adjusted C-index = 0.749

Isolated cases: anotia/microtia with no other structural anomaly diagnosis, non-isolated cases: anotia/microtia with the presence of other structural anomalies (excluding chromosomal anomalies), NS, not specified; OR, odds ratio; aOR, adjusted odds ratio; CI, confidence interval.

^a^ Adjusted for pregnancy BMI, age, education, folic acid, gestational diabetes, smoking, alcohol intake, annual household income, and study center.

^b^ Adjusted for maternal age, race/ethnicity, border residence, maternal birthplace, maternal education, year of infant birth, and infant sex.

^c^ Adjusted by sex, maternal age, hospital, and year of birth.

^d^ Adjusted by gender, age, region, syndrome, and family history.

^e^ Risk factor prevalence.

**Table 6:** Risk factors for microtia/anotia compared by all, isolated, and non-isolated cases.

Risk factors for microtia/anotia	OR (95% CI)		
	All cases	Isolated cases	Immunization before pregnancy
Shaw, 2004 (36)	NR	(*n* = 160)	(*n* = 389)
White, non-Hispanic		Reference	Reference
U.S. born Hispanic		4.6 (2.4–9.1)^a^	2.0 (1.4–2.9)^a^
Foreign born Hispanic		6.5 (3.5–12.1)^a^	1.8 (1.3–2.5)^a^
Asian		3.2 (1.4–7.4)^a^	NR
Maternal age 30–34 (vs. 20–24)		NR	1.4 (1.0–1.9)^a^
Male infant (vs. female)		NR	1.2 (1.0–1.5)^a^
Education >12 years (vs. <12 years)		NR	0.6 (0.4–0.8)^a^
Ma, 2010 (22)	(*n* = 410)	(*n* = 297)	NR
No intake	Reference	Reference	
Periconceptional vitamin (Folic acid) intake	NR	0.69 (0.49–0.98)^b^	
Non-obese women periconceptional vitamin (Folic acid) intake	0.63 (0.44–0.91)^b^	0.51 (0.34–0.77)^b^	
Ma, 2012 (23)	(*n* = 368)	(*n* = 273)	(*n* = 95)
Adequate intake	Reference	Reference	Reference
Low carbohydrate intake	1.59 (1.07–2.38)^c^	1.73 (1.09–2.73)^c^	NR
Low glycemic load	1.48 (1.00–2.19)^c^	NR	NR
High cysteine intake	NR	NR	2.12 (1.09–4.13)^c^
Low folate intake	1.57 (1.09–2.25)^c^	1.55 (1.02–2.36)^c^	NR
Van Bennekom, 2013 (25)	(*n* = 411)	(*n* = 296)	NR
No exposure	Reference	Reference	
NSAIDs	1.3 (1.0–1.8)^d^	1.2 (1.0–1.6)^d^	
Maternal diabetes	3.4 (1.3–8.5)^d^	7.2 (3.9–13.1)^d^	
Pre-existing Hypertension	NR	1.6 (1.0–2.5)^d^	
Multiple gestation	NR	2.5 (1.5–4.2)^d^	
Deng, 2016 (39)	(*n* = 1,933)	(*n* = 1,419)	(*n* = 514)
Male infant (vs. female)	1.29 (1.18–1.41)^e^	1.38 (1.24–2.53)^e^	NR
Maternal urban residence (vs. rural)	1.19 (1.08–1.32)^e^	1.29 (1.15–1.46)^e^	NR
Maternal age 20–24	Reference	Reference	Reference
Maternal age: 30–34	1.18 (1.03–1.36)^e^	1.20 (1.03–1.41)^e^	NR
Maternal age ≥35	1.42 (1.18–1.72)^e^	1.26 (1.01–1.57)^e^	1.92 (1.39–2.62)^e^
Ryan, 2019 (29)	(*n* = 699)	(*n* = 480)	(*n* = 219)
Male infant (vs. female)	1.29 (1.10–1.50)	1.36 (1.13–1.64)	NR
Multifetal gestation (vs. singleton)	1.68 (1.16–2.42)	NR	2.67 (1.58–4.49)
Gestational age >37 weeks	Reference	NR	Reference
Gestational age <32 weeks	3.63 (2.42–5.45)	NR	10.24 (6.24–16.80)
Gestational age 32–36	2.46 (1.99–3.03)	NR	5.45 (4.02–7.40)
BMI ≥ 30 (vs. 18.5–25)	NR	NR	1.65 (1.18–2.31)
No history of diabetes	Reference	Reference	Reference
Type I diabetes	9.89 (5.46–17.92)^f^	4.93 (1.99–12.18)^f^	23.48 (12.03–45.83)^f^
Type II diabetes	4.70 (2.56–8.63)^f^	NR	13.91 (7.17–26.96)^f^
Gestational diabetes	NR	NR	1.62 (1.04–2.52)^f^
No drinking and no smoking	Reference	NR	Reference
Binge drinking	1.84 (1.06–3.21)	NR	NR
Smoking ≥5 cigarettes daily	NR	NR	1.70 (1.12–2.59)^f^
Schraw, 2023 (33)	(*n* = 1,322)	(*n* = 982)	(*n* = 340)
Male infant (vs. female)	1.31 (1.17–1.46)^g^	1.34 (1.18–1.53)^g^	2.73 (1.87–3.99)^g^
White, non-Hispanic	Reference	Reference	NR
Hispanic	2.90 (2.48–3.39)^g^	2.89 (2.41–3.46)^g^	NR
Non-Hispanic other	1.72 (1.27–2.33)^g^	1.67 (1.18–2.38)^g^	NR
Black, Non-Hispanic	0.55 (0.39–0.76)^g^	0.41 (0.27–0.64)^g^	NR
>High school education	Reference	Reference	NR
<High school education	1.25 (1.08–1.45)^g^	1.29 (1.09–1.54)^g^	NR
No history of diabetes	Reference	Reference	Reference
Maternal diabetes	2.0 (1.64–2.44)^g^	1.35 (1.03–1.76)^g^	4.53 (3.21–6.40)^g^
Pregestational diabetes	5.13 (3.59–7.33)^g^	NR	12.9 (7.8–21.4)^g^
Gestational diabetes	1.66 (1.27–5.15)^g^	NR	NR
Maternal age 30–39 (vs. 20–29)	1.13 (1.01–1.28)^g^	1.27 (1.10–1.47)^g^	NR
Multiple births (vs. singletons)	NR	NR	2.48 (1.46–4.19)^g^
Residence in border county	NR	0.83 (0.70–0.99)^g^	NR

Isolated cases: anotia/microtia with no other structural anomaly diagnosis, non-isolated cases: anotia/microtia with the presence of other structural anomalies (excluding chromosomal anomalies), OR, odds ratio; aOR, adjusted odds ratio; CI, confidence interval; NR, not reported.

^a^ Adjusted for maternal race/ethnicity, education, age, parity, plurality, and infant sex.

^b^ Adjusted for maternal race/ethnicity, education, and study site.

^c^ Adjusted for maternal race/ethnicity, education, intake of supplements containing folic acid, fertility treatment, study site and total energy intake.

^d^ Adjusted for maternal race/ethnicity, education, periconceptional folic acid use, and study center.

^e^ Adjusted for geographic area, maternal residence, maternal age, and infant sex.

^f^ Adjusted for maternal education, birthplace, and race/ethnicity.

^g^ Adjusted for factors associated with the index outcomes at *p* < 0.05 in univariable models.

Risk factors by demographics and parental health behaviors include:

(a) Parental age. One study reported that Hispanic mothers have higher risk of congenital ear malformations, compared to non-Hispanic white mothers for almost all age groups (26). Conversely, maternal, and paternal age ≥30 was associated with increased odds of microtia/anotia compared to those of younger age in two studies (27, 38), and in three studies, mothers of >30 years compared to mothers <30 years for isolated, non-isolated, and all cases of microtia/anotia (33, 36, 39). Another study reported that mothers of 25–29 years have a higher risk compared with control group (32). And one that there is a statistically significant difference (*p* < 0.01) between maternal mean age compared to controls (45).
(b) Maternal race/ethnicity. Multiple studies reported the risk association with maternal race/ethnicity, showing an increased risk for Hispanics compared to other groups (26, 31), and for isolated, non-isolated and all cases combined (33), and one also reporting increased risk in Asians for isolated cases (36). Some studies reported that African Americans have significant less risk of external ear malformations compared with other groups (26, 31), and one compared to with isolated and all cases of microtia/anotia (33).
(c) Parental education. Less educated parents (high school or less/<12 years of education) showed higher risk of having a newborn with congenital ear malformations in four studies (21, 32, 33, 38), one for Hispanic mothers compared to non-Hispanics regarding educational level (26), and other a decreased risk for mothers with ≥12 years of education (36).
(d) Maternal employment and household income. One study reported a greater risk for maternal employment outside of home, compared to housewives (24), and one for household income ranging from <10,000 to ≥40,000 (USD) for Hispanics compared to non-Hispanic whites (26).
(e) Parental residential area and altitude. One study reported a higher risk for external ear malformations in patients with urban residency compared with those with rural residency (42), other showed a greater risk for isolated cases of microtia/anotia (39) and one reported in risk for urban residency (35), while other reported the opposite (21). Only one study reported moderate altitude (1,511–2,426 m) as a risk factor, compared with lower altitudes [<1,499 m] (34), and one a decreased risk for mothers living in U.S.A-Mexico border counties, compared to other U.S.A counties (33).
(f) Parental chemical exposure. One study reported an association between chemical exposure such as formaldehyde, pesticides, and organic solvents, during the first trimester of pregnancy, and a significantly higher risk of having a child with severe microtia/atresia; medicines like progesterone; traditional Chinese medicines, such as *radix isatidis*, pseudo-ginseng and goldthread root; and NSAIDs (nonsteroidal anti-inflammatory drugs), were also significantly associated (27). Four studies showed that congenital ear malformations increase with parental chemical exposure, including heavy metals, dust, and SO_2_ exposure during the 3 months before conception and the 3 months after conception (21, 28, 30, 32), two reported environmental pollution as a risk factor for congenital microtia (21, 30). The association between parental drinking and smoking habits were reported by multiple studies; one study showed an association with alcohol and congenital microtia compared with non-drinkers (35), two with smoking ≥1 cigarette a day compared to non-smokers (24, 30), and one for smoking 1 month before to 3 months after conception compared to non-Hispanic whites (26). One reported increased risk only for binge drinking, associated with all cases of microtia/anotia, and smoking ≥5 cigarettes daily for non-isolated cases (29). Finally, only one study showed a significant risk increase of congenital microtia with pet contact during pregnancy (21).
(g) Family history of congenital ear malformations. Two studies showed a greater risk for congenital ear malformations on those with family history of malformations (20, 24).

Risk factors by pregnancy characteristics and parental clinical features, are shown in Table 5, and include:

(a) Infant sex. Multiple studies reported higher risk of congenital ear malformations in male infants compared to females (32, 38, 41, 42, 44, 45). Some reported similar results but comparing between isolated, non-isolated, and all cases combined of microtia/anotia (29, 33, 36, 39). While only one study showed increased risk for both male and females for U.S. born and foreign-born Hispanics compared to non-Hispanics (26).
(b) Gestational age and weight. Four authors showed increased risk of congenital external ear malformations for premature newborns compared to full-term newborns (31, 32, 37, 44), and one for premature (32–36 weeks), and very premature (<32 weeks) newborns for non-isolated and all cases of microtia/anotia (29).
(c) Maternal BMI and gestational diabetes. One study compared the risk association of pregnancy BMI and gestational diabetes for isolated microtia/anotia in U.S. born and foreign-born Hispanics compared to non-Hispanic whites, showing risk for all of the maternal BMI groups, and a greater risk for those with BMI ≥30 (26), one study for BMI ≥30 compared to a BMI of 18.5–25, and gestational diabetes for isolated cases (29), and one of maternal diabetes for isolated and all cases combined, with a greater risk for non-isolated cases, and gestational diabetes for all cases (33).
(d) Parental chronic diseases. Two studies reported an increased risk for microtia/anotia with maternal and paternal chronic diseases (30), and maternal chronic diseases (24), although no specific illness is mentioned, and one of mothers with chronic diabetes (31). One study reported higher risk for microtia/anotia with maternal diabetes (type I and II) in isolated and all cases of microtia/anotia, and with pre-existing hypertension with isolated cases (25), other showed increased risk with type I diabetes for all cases, including isolated and non-isolated, while type II increased the risk significantly only for non-isolated cases (29), and one study for maternal diabetes and pregestational diabetes for all cases, isolated and non-isolated, and for non-isolated and all cases respectively (33).
(e) Maternal medication and nutrition. Six studies showed a significant risk for external ear malformations in mothers taking medication during pregnancy, including analgesics (NSAIDs), antibiotics, antiemetics, antihypertensives, antispasmodics, oral contraceptives, and traditional Chinese medicine (21, 24, 25, 27, 30, 35). One showing that low folate intake increased the risk for isolated and all cases of microtia (23), and one showing higher risk in U.S. and foreign born Hispanics, than non-Hispanic white population (26); conversely, adequate periconceptional folate intake showed a marked reduction of risks in two studies (28, 30), and one showed a risk reduction for isolated cases, isolated cases and all cases for non-obese women, without significant results in non-isolated cases (22).
(f) Pregnancy complications and infections. Three studies reported greater risk for external ear malformations of mothers with abnormal pregnancies, including vaginal bleeding and anemia during the first trimester (20, 27, 30). Seven studies reported a significant increase of congenital external ear malformations with maternal infections during pregnancy (21, 24, 27, 28, 30, 31, 43), particularly in those presenting with viral infections and cold-like symptoms during pregnancy (24, 27, 28, 30, 43), and only one reported rubella vaccination before pregnancy to have a protective effect (35).
(g) Miscarriages. Two studies showed the risks of having an infant with congenital external ear malformations with threated abortion (30, 35), two with history of spontaneous abortion (28, 43), and one with both threatened abortion and history of miscarriages (27).
(h) Multiple pregnancies. Four studies showed that the risk of congenital ear malformations increased with multiple pregnancies and deliveries compared with primiparas (24, 30, 32, 37), and one showed an increased risk for non-singletons compared to singletons (31). One study reported higher risk of non-singletons for non-isolated and all cases of microtia/anotia (29), one only for isolated cases (25), and another only for non-isolated cases (33).
(i) Finally, one study compiled a predictive nomogram for maternal age, history of miscarriages, viral infections, anemia, progesterone use, paternal alcohol use, and topography of resident areas (40).

Table 7 summarizes some of the most reported defects and syndromes associated with external ear malformations from the included studies. The most common deformations associated with non-isolated cases of external ear malformations were cleft lip/palate, congenital heart defects, musculoskeletal deformities of skull, face and jaw, and preauricular tags and fistulas (25, 28, 33, 35, 36, 40, 43, 45). The multiple syndromes reported, include trisomy 18, 13, 21, Treacher-Collins, Nager syndrome, EEC syndrome, 4p- (Wolf-Hirschhorn) syndrome, X-linked dominant chondrodysplasia punctata, 22q11.2 deletion syndrome, and OAVS (25, 35, 37, 38, 40, 43, 45).

**Table 7:** Associated defects and syndromes with external ear malformations by study.

Associated defects and syndromes	n (%)
Shaw et al. (36) Dermatoglyphic and other skin anomalies Skull/face bones anomalies Choanal atresia and other nose anomalies Other ear anomalies Musculoskeletal deformities of skull, face, and jaw	*n* = 389* 173 (44.4) 165 (42.4) 113 (29.1) 112 (28.8) 91 (23.4)
Forrester and Merz (37) Trisomy 18 Trisomy 21 Trisomy 13 Chromosome 9 deletion	*n* = 9* 4 (44.4) 3 (33.3) 1 (11.1) 1 (11.1)
Canfield et al. (38) Trisomy 21 Trisomy 18 Trisomy 13 Treacher-Collins Unknown syndromes	*n* = 121* 6 (4.9) 16 (13.2) 12 (9.9) 19 (15.7) 38 (31)
Wu et al. (43) Hemifacial microsomia Preauricular tags Cleft lip/palate Congenital heart problems Preaxal polydactyly	*n* = 150* 131 (37.97) 87 (25.21) 5 (1.45) 2 (0.58) 2 (0.58)
Lee et al. (35) Hemifacial microsomia Preauricular skin tags Cleft lip/palate Other	*n* = 131* 107 (28.6) 45 (12) 6 (1.6) 4 (0.8)
Yamauchi et al. (45) Congenital heart disease Cleft lip/palate Vertebral defects Anomalies of extremities Individual cases Treacher-Collins syndrome Nager syndrome EEC syndrome 4p- (Wolf-Hirschhorn) syndrome X-linked dominant chondrodysplasia punctata 22q11.2 deletion syndrome 21 monosomy	*n* = 57* 20 (5) 16 (4) 7 (2) 7 (2)
Van Bennekom et al. (25) OAVS and cardiac defects OAVS, cardiac defects and hydrocephaly OAVS and cleft lip/palate OAVS, sacral agenesis and cardiac defects Microtia, sacral agenesis, cardiac and central nervous system defects Microtia, cleft palate, cardiac defects, and limb deficiency Microtia, cardiac defects and either sacral agenesis, clubfeet, or hydrocephaly Microtia and cardiac defects	*n* = 14* 3 (21.4) 1 (7.1) 1 (7.1) 1 (7.1) 1 (7.1) 1 (7.1) 3 (21.4) 3 (21.4)
Li et al. (28) Mandibular dysplasia Pre-auricular fistulas or tags Eye defects Tragus defects Cleft lip/palate Cardiac defects Other defects (polydactyly, spinal defects, and anosmia)	*n* = 278* 168 (18.4) 80 (8.78) 23 (2.52) 17 (1.87) 10 (1.10) 4 (0.44) 9 (0.99)
Guo et al. (40) Hemifacial microsomia Pre-auricular tags Facial paralysis Congenital heart defects Pre-auricular fistula Hemifacial tags Macrostomia Mandibular micrognathia Epibulbar dermoid Orbital hypertelorism Other defects (strabismus, blepharoptosis, scoliosis, duplex kidney and iridocoloboma)	*n* = 392* 335 (34.7) 248 (25.7) 106 (11.0) 85 (8.8) 55 (5.7) 29 (3.0) 29 (3.0) 17 (1.8) 11 (1.1) 11 (1.1) 20 (2)
Schraw et al. (33) Central nervous system deformities Eye deformities Cardiovascular deformities Cleft lip/palate Urinary deformities Musculoskeletal deformities	*n* = 340* 50 (14.7) 20 (5.9) 214 (62.9) 43 (12.6) 81 (23.9) 51 (15)

* Some cases presented multiple defects/malformations. OAVS, oculo-auriculo-vertebral spectrum.

## 4 Discussion

This review aimed to compile and analyze the literature describing external ear congenital anomalies occurrence, characteristics, and associated factors. Congenital anomalies of the external ear vary widely in type and severity, while severe malformations such as anotia/microtia are uncommon. The frequency reported by the studies in this review ranged from 0.21 to 4.34 cases per 10,000 live births (36, 38, 46), higher than the reported by the literature of 0.8 to 2.4 per 10 000 live births (39, 47, 48). This variation might be due to inclusion criteria or higher rates reported in certain regions in comparison with other countries.

The studies reviewed show that external ear anomalies are more frequent in males, usually unilateral, more often in the right ear, Hispanic and Asian populations show higher prevalence compared to other ethnicities. Paternal and maternal age, environmental and pharmacological exposures, pregnancy, or perinatal related complications have been also associated with congenital ear malformations. From the studies presented in this review, twenty-three reported a frequency ranging from 47.1% to 78.5% for males (20, 21, 24–33, 35–45). This is consistent with other studies reporting male predominance: 68.6% in China (27), 58% in Finland (46), 60% in the United States (29, 36, 38, 49) and 54% in Hungary (50). It is worth noticing that both male and female, show similar severity grades (27, 49).

The reports included in this review show that 84% of external ear anomalies are unilateral, and mostly on the right ear (20, 24, 27, 28, 33, 35, 37, 40, 43, 45), consistent with the information presented in other studies (1, 51–53). The mechanisms for this predominance are still unclear; some authors have hypothesized that the greater prevalence of unilateral microtia cases may be related to a localized effect during embryogenesis, resulting in occlusion of a single vessel and thus, causing unilateral alterations (54). This has been attributed to a reduced or complete loss of blood flow to pharyngeal arches, resulting in hypoxia and damage to normal tissue (55), due to the interruption of blood flow to previously formed tissue, vasoconstriction, or underdevelopment of the arterial system required for adequate blood supply to the developing tissues (56). However, epidemiological, or experimental data are insufficient to support this hypothesis; and furthermore, even malformations caused by genetic alterations occur unilaterally, thus other factors acting through nonvascular mechanisms should be considered (55).

From the studies reporting ethnicity, nine found a higher prevalence among Hispanics compared to Caucasians and African Americans in the United States (22, 23, 25, 26, 29, 31, 33, 36, 38). Population-based studies have shown that the prevalence of external ear anomalies is more common in Asians, Hispanic, and Native American population, than in African American and Caucasian populations (57–59). This has been widely reported in studies from Mexico, Paraguay, China, Argentina, Chile, Ecuador, and Japan, where the prevalence ranges from 0.14 to 17.4 per 10,000 births (60–66), compared to studies from England, Italy, France, Hungary, and the United States of America, where the prevalence ranges between 0.66 to 4.34 per 10,000 births (36, 38, 50, 65–73). It is worth considering that prevalence rates are often calculated from live-births records, thus they might be underestimated in populations with high rates of stillbirths and abortions. Furthermore, several factors may relate to ethnicity differences in prevalence. For instance, the higher prevalence rate for Hispanic population in this review, for both US-born and Latin-American Hispanics (22, 23, 25, 26, 29, 31, 33, 36, 38) could be related to cultural behavior, regional differences, genetic variations, and environmental factors, such as socioeconomic status, nutrition, and prenatal care, or a combination of all (33, 56).

Studies report multiple environmental and demographic factors, that may be associated with external ear anomalies: parental age, education level, maternal employment, household income, residential area, altitude, environmental/chemical exposure during the three months before or after conception, drinking and smoking have shown to increase the risk of developing external ear malformations (21, 24–36, 38–40, 42, 45). Altitude has an important impact on external ear malformations in some countries in South America. For instance, there is a higher prevalence for microtia in Quito, Ecuador; La Paz, Bolivia; and Bogota, Colombia, that have higher altitude compared to other countries in South America (74–76). This is supported by the evidence of increased circulating levels of catecholamines and inflammatory cytokines during pregnancy, resulting in damage to the developing embryo and in intra-uterine growth restriction, and increased frequency of preeclampsia and stillbirths, in populations living at high-altitude (77, 78). Pregnancy and perinatal characteristics, as well as parental clinical features have shown to increase the risk of developing external ear anomalies. Among them: male sex, prematurity, low gestational weight, high maternal BMI, gestational diabetes, chronic illness, viral infections (common cold and influenza), some medications (NSAIDs, antihypertensives, antiemetics, progesterone, traditional Chinese medicines), low periconceptional vitamin intake, previous history of threatened abortion, bleeding, and anemia during the first trimester of pregnancy (20–33, 35–38, 40–45). Several risk factors differed between isolated and non-isolated cases of microtia/anotia. In the reviewed articles, the risk factors reported in isolated cases included: being of Asian and Hispanic descent (33, 36), low carbohydrate and folate intake (23), NSAIDs use, pre-existing maternal hypertension, multiple gestations (25), maternal education below high school (33), and urban residency (39); and protective factors were periconceptional folic acid intake (22) and maternal residence in border counties (33). Risk factors reported in non-isolated cases included: high cysteine intake (23), smoking ≥5 cigarettes daily, prematurity, BMI ≥30 (29), multiple births (33), and pre-gestational and gestational diabetes (29, 33); and maternal education above 12 years was reported as a protective factor (36). Notably, maternal diabetes has been consistently linked to a slight but significant increase in the risk of non-isolated cases of microtia/anotia by multiple studies (25, 31, 79–81).

These factors might disrupt the very complex sequence in the development of the external ear, but the mechanisms are not fully understood. This sequence can also be disrupted by mechanical trauma; as the external surface of the developing embryo is in close contact with the uterine wall, where increased tissue fragility or reduced cell-specific adhesiveness may increase the embryos susceptibility to physical or mechanical trauma (82), or in local vascular disruptions and transient focal tissue ischemia (56).

Some genetic studies have shown possible associations between gene mutations and their effect on the pharyngeal arches and external ear malformations. One of the most studied is *HOXA2*, an important transcriptional regulator for ear development (83, 84), suggesting that *HOXA2* may be fundamental in orchestrating the auricle morphogenesis (56, 85, 86). Furthermore, twin studies, particularly those monozygotic with shared genotype, have also demonstrated a strong genetic association (87), suggesting that apart from environmental factors and behavioral factors, these malformations may also be linked genetically.

Multiple pregnancies have been reported to increase the risk of external ear malformations (24, 25, 29–33, 37), and more frequently in those by assisted reproduction techniques (44). Studies of *In-vitro Fertilization* (IVF) and *intracytoplasmic sperm injection* (ICSI) compared with those conceived naturally have shown a significantly higher risk of congenital malformations (88, 89), including eye, ear, face, and neck malformations (89–91). It has been reported that the techniques used with these procedures, such as medications used to induce ovulation and/or to maintain the pregnancy, culture media composition, length of time in culture, the freezing of embryos, among others, may be involved (92, 93). However, the specific ear malformations were not mentioned in these studies, thus further research is needed to define the type of ear malformations that are associated with these techniques.

Other factors such as consanguinity have been studied, with some studies reporting cases of autosomal-recessive and non-syndromic forms of bilateral microtia in consanguineous families (94, 95) However, the information about this topic is limited, and the association with external ear anomalies is still unclear.

Congenital external ear malformations may occur as an isolated defect, or with other defects and syndromes. Cleft lip/palate, congenital heart defects, musculoskeletal deformities of skull, face and jaw, and preauricular tags and fistulas were the most reported malformation associated with congenital external ear malformations in non-isolated cases (28, 35, 40, 43), as reported from multiple studies (35, 43, 44, 96). Treacher-Collins, trisomy 18, 13 21, and OAVS were the most reported syndromes (25, 37, 38, 45). It is important to note that many syndromes may not be reported due to the requirements for a karyotype to establish a diagnosis (37), and for those with known diagnosis, certain external ear malformations, such as milder forms of microtia, may be wrongly classified or not reported (38).

External ear malformations may relate to hearing loss, particularly in bilateral cases, justifying routine newborn hearing screening for early detection and prompt treatment (6). Surgical corrections of external ear malformations require a wide understanding of ear anatomy and its development (10). Over the years many techniques have been used and modified to accomplish optimal functional and aesthetic results. The type of technique depends on the presented malformation and whether the malformation is bilateral or unilateral (97). Many surgical techniques include cartilage sculpting from autogenous costal cartilage, and the use of sutures to reshape the ear (10, 17, 97, 98). Several complications can occur with these techniques, ranging from restenosis and otorrhea, to facial nerve injury, cartilage fracture, ear deformations, tympanic membrane perforation and inner ear trauma, worsening hearing impairment (10, 17, 97, 99).

Non-surgical techniques have proven to be highly effective in correcting minor malformations and deformations (such as deformities of the conchal crus, helix, Stahl ears, lidded ears, and prominent ears). Some of the most described techniques include ear molding techniques (7, 10, 100, 101), or laser assisted cartilage reshaping (102), offering an effective approach that can provide optimal results without the need for invasive procedures (7, 101, 102).

Missed diagnoses of congenital external ear malformations can result in delayed intervention and thus, speech developmental delays. Hearing screening in newborns has been reported as the most efficient method for early detection of hearing impairment (6). Once a diagnosis has been made, it is important to carefully select patients, according to strict criteria, including age and absence of sensorineural components of hearing loss, to avoid cases on where surgical intervention may not show favorable results (17, 99).

Overall, the results of this review describe several factors associated with congenital external ear anomalies. However, it has some limitations. There are few studies on external ear malformations, most of them focused on anotia/microtia only. Furthermore, the information regarding race/ethnicity predisposition is limited to a few populations, where the cultural aspects may go under-reported. Moreover, no studies were found of these anomalies in fetuses, thus the reports are limited to registered live births. Nevertheless, this review shows the complexity surrounding the external ear development and some of the associated factors that can result in its malformations.

In conclusion, congenital external ear anomalies include a wide variety of malformations that can occur isolated or associated with other malformations or syndromes. It is important to take environmental, cultural, and social aspects into consideration as a possible explanation for the wide variation across populations. External ear anomalies may cause conductive hearing loss, especially in bilateral cases, since the ear structure aids in the transmission of sound to the middle and inner ear and, depending on their type and severity, can lead to speech impediments. This highlights the importance of an early detection, classification, and repair to avoid childhood disability.

## Data Availability

The original contributions presented in the study are included in the article/Supplementary Material, further inquiries can be directed to the corresponding author.
